# Use and Abuse of the Speculum

**Published:** 1850-11

**Authors:** 


### USE AND ABUSE OF THE SPECULUM.

The recent attacks of Professor Tyler Smith, of Dr. Robert Lee, and of Marshall Hall, upon the views of Dr. James Henry Bennet, contained in Dr. Bennet's work on the Uterus, have called forth from the latter gentleman a scathing rejoinder. The work of Dr. Bennet is one for which we entertain a great deal of respect; it is a work of an eminently practical character, and one that has already done good service for the progress of medicine, and is destined to command confidence among medical men, as long as it may be studied. The attacks made by Drs. Smith, Lee, and Hall were not conceived in a very gentle, nor a very philosophical spirit, and displayed more of the sharpness of the partisan, than devotion to truth.

We think, however, that Dr. Bennet has given Drs. Smith, Lee, and Hall a series of knotty arguments, in return for their gratuitous assaults, and Dr. Bennet will be likely to carry the great mass of the profession with him. He is worthy of confidence, and may confidently rely upon obtaining and retaining it. B.

